# Factors associated with secondhand smoke incursion into the homes of non-smoking residents in a multi-unit housing complex: a cross-sectional study in Seoul, Korea

**DOI:** 10.1186/s12889-017-4774-x

**Published:** 2017-09-25

**Authors:** Jeonghoon Kim, Kiyoung Lee, KyooSang Kim

**Affiliations:** 1 Department of Environmental Health Research, Seoul Medical Center, 156 Sinnae-ro, Jungnang-gu, Seoul, 02053 Republic of Korea; 2 Department of Environmental Health Sciences, Graduate School of Public Health, Seoul National University, 1 Gwanak-ro, Gwanak-gu, Seoul, 08826 Republic of Korea; 3 Institute of Health and Environment, Graduate School of Public Health, Seoul National University, 1 Gwanak-ro, Gwanak-gu, Seoul, 08826 Republic of Korea

**Keywords:** Incursion, Multi-unit housing, Nonsmoker, Resident, Secondhand smoke, Smoke-free rule

## Abstract

**Background:**

In a multi-unit housing (MUH) complex, secondhand smoke (SHS) can pass from one living space to another. The aim of this study was to determine the prevalence of SHS incursion, and to establish the relationship between SHS incursion and socio-demographic and built environmental factors in MUH in Korea.

**Methods:**

A population-based sample of 2600 residents (aged ≥19 years) living in MUH from across the city of Seoul, Korea, was obtained through a web-based selection panel. The residents completed a questionnaire detailing socio-demographic factors, smoking status, frequency of SHS incursion, and built environmental factors. The presence of a personal smoke-free home rule was determined by residents declaring that no one smoked inside the home.

**Results:**

Of the 2600 participants, non-smoking residents who lived in homes with a personal smoke-free rule were selected for further analysis (*n* = 1784). In the previous 12 months, 74.7% of residents had experienced SHS incursion ≥1 times. A multivariate ordinal logistic regression analysis indicated that residents who spent more time at home, lived with children, supported the implementation of smoke-free regulations in MUH, lived in small homes, lived in homes with natural ventilation provided by opening a front door or the windows and front door, and lived in homes with more frequent natural ventilation were more likely to report SHS incursion into their homes.

**Conclusions:**

The majority of the non-smoking residents experienced SHS incursion, even with a personal smoke-free rule in their homes. A smoke-free policy in MUH is needed to protect residents from SHS exposure when they are at home.

**Electronic supplementary material:**

The online version of this article (10.1186/s12889-017-4774-x) contains supplementary material, which is available to authorized users.

## Background

Secondhand smoke (SHS) exposure is causally linked to cardiovascular disease, respiratory effects, and lung cancer [1–4]. Exposure to SHS in children is associated with increased risks of asthma, middle ear infections, and sudden death syndrome in infancy [4]. SHS exposure caused 603,000 premature deaths in 2004, equivalent to 1.0% of worldwide mortality, based on data from 192 countries [5]. The US Surgeon General concluded that there is no risk-free level of SHS exposure and only the elimination of indoor smoking can protect non-smokers [4].

The extensive evidence of adverse health effects associated with SHS exposure has led many countries to introduce smoke-free regulations in indoor public spaces and work places. The implementation of smoke-free regulations has resulted in an improvement in indoor air quality [6, 7] and the health of non-smoking staff in hospitality venues [8, 9]. However, there has been a limited implementation of similar regulations in personal living spaces. Although it might be difficult to pass legislation to restrict smoking in a private home, public housing could be smoke-free. Smoke-free public rule of the U.S. Department of Housing and Urban Development went into effects since February 3, 2017 [10]. Public Housing Authorities were required to adopt and implement a smoke-free regulation in all of their public housing properties by August 2018.

Residents living in multi-unit housing (MUH) are particularly susceptible to SHS exposure because SHS can be transferred between units in MUH [11]. In 2009, 44.0–46.2% of Americans who lived in smoke-free MUH reported SHS incursion into their units [12]. In Denmark, 28.2% of MUH residents living in non-smoking homes reported that SHS from their neighbors had seeped into their homes [13]. In Hong Kong, 11.8% of students who lived in homes without smokers were experienced SHS in their homes that came from neighboring flats [14]. Because people spend the majority of their time in their homes, SHS exposure at home can be a significant contributor to their total SHS exposure [4].

The prevalence of SHS incursion in MUH in Korea has not been established. Furthermore, most of the studies that have been conducted have examined the relationship between SHS incursion into MUH living spaces and socio-demographic factors. Smoking status, the presence of children living in the home, and the type of MUH have been identified as predictors of SHS incursion [13, 15, 16]. A previous study reported that up to 65% of the air in a private unit could come from somewhere else in the building depending on the construction and age of building [17]. The aim of this study was to determine the prevalence of SHS incursion in MUH and to establish the relationship between SHS incursion into the homes of non-smoking residents and socio-demographic and built environmental factors.

## Methods

### Sample

**Fig. 1:**
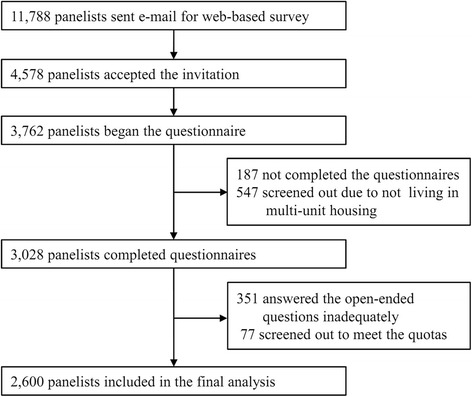
Flow chart toward the final study sample

The study was approved by Seoul Medical Center’s institutional review board (IRB No. 2015–051). Because we used a web-based survey using internet panelists who voluntarily enrolled in the survey company, written informed consent of the panelists was not necessary. The study included internet panelists (≥19 years) who lived in MUH in Seoul, Korea. The MUH in the study included apartments and attached homes. In Korea, an apartment is defined as a unit in a building with five or more stories, similar to a high-rise condominium building in the US. An attached home is a unit in a multi-family building less than five stories tall. Data were collected from 21 August to 4 September 2015. Using August 2015 population statistics from the residential registry of the Ministry of the Interior (MI) [18], quotas were calculated for sex, age, and residential region that corresponded to the Seoul population. Although the proportion of residents in the various categories differed between apartments and attached homes, we considered that about 50% of each category was present in each type of residence, enabling us to determine whether housing type played an important role in SHS incursion. A flow chart describing the selection of final study sample is shown in Fig. 1. Of the more than 300,000 panelists, 11,788 people were selected for the study because they had participated in web-based survey within the previous 12 months. Of these 4578 accepted the invitation to participate, 3762 began the questionnaire. Of these 3762 residents, 187 did not complete the questionnaire, and 547 were screened out because they did not live in MUH. Thus, a total of 3028 residents completed the questionnaire and were evaluated further. Of these 3028 residents, 351 answered the open-ended questions inadequately and were excluded, and a further 77 were screened out to meet the quotas. Ultimately, 2600 residents were included in the final analysis.

The initial sample size that needed to provide 95% confidence intervals (CI) with a margin of error of 0.03 was calculated to be 1067. Because non-smoking homes accounted for 41–51% of all MUH units [19], we collected more samples than our required initial sample size.

### Socio-demographic factors

The self-reported socio-demographic factors investigated were sex, age, household income, education, housing type, time spent at home, number of residents, children aged ≤18 years living in home, type of ownership, duration in current residence, presence of other smokers inside home (i.e., family members or regular visitors), support for the implementation of smoke-free regulations in MUH, and living in a home with a personal smoke-free rule (See Additional file 1: Table S1 for the detailed). Respondents were determined to be living in a home with a personal smoke-free rule if they indicated that they lived in a home in which no one smoked inside. Therefore, the homes with a personal smoke-free rule included homes without smokers or homes with smokers, but smokers were not allowed to smoke inside homes.

### Smoking status

Residents were asked whether they were currently smoking “every day,” “sometimes,” “in the past but not currently,” or “never.” Residents were classified as non-smokers if they reported smoking “in the past but not currently” or “never.” (See Additional file 1: Table S2 for the detailed).

### Frequency of SHS incursion

Residents were asked how often they could smell tobacco smoke that entered their living space from somewhere else in or around their building during a 12-month period (See Additional file 1: Table S3 for the detailed). The possible responses were “never,” “once a month or less,” “twice a month,” “four times a month,” “two to four times a week,” or “every day.” A similar question was used in a previous study [15]. When a resident indicated that they had experienced SHS incursion within the previous 12 months, we asked them where the SHS had entered and gave them the following options: “balcony,” “window,” “bathroom,” “front door,” or “other location.”

### Built environmental factors

Residents were asked to identify various built environmental factors in the MUH. The environmental factors investigated were date of construction, type of corridor, home size, presence of balcony, presence of air conditioning, method of natural ventilation, and the frequency of natural ventilation (See Additional file 1: Table S4 for the detailed). Date of construction might be associated with SHS incursion because air that contained SHS particles could be infiltrated from other unit or the building envelope [17, 20]. Other factors might be associated with SHS incursion due to resident’s behavior at homes (e.g., method and frequency of natural ventilation) [21].

### Statistical analysis

For the statistical analyses, the self-reported frequency of SHS incursion in MUH was classified into four ordinal categories (never or ≤1, 2–4, or >4 times/month); similar proportions were found in all categories. A chi-square test was used to compare residents who were smokers and non-smokers according to socio-demographic factors and the frequency of SHS incursion. The Cochran-Mantel-Haenszel test was used to select potential socio-demographic and built environmental factors on SHS incursion. Using the variables identified in the Cochran-Mantel-Haenszel test (*p* < 0.05), ordinal logistic regression analysis was used to assess the relationships between SHS incursion and the variables. The score test for the proportional odds assumption in the ordinal regression models was conducted to confirm or reject the assumption. When the assumption was violated (*p* < 0.05), partial proportional odds model was fit. Odds ratios (ORs) for the variables in the model were reported with a 95% CI. A *p-*value 0.05 was considered significant in all analyses. SAS 9.2 software (SAS Institute, Inc., Cary, NC, USA) was used for all statistical analyses.

## Results

The distributions of sex, age, and residential region in the Seoul population obtained from the MI [18] and the population in this study are shown in Table 1. The distributions of sex and residential region in the study population were similar to those of the Seoul population. The study population was slightly younger, on average, than the Seoul population.

**Table 1:** Distributions of sex, age, and residential region in Seoul and study population

	Seoul population (%; *n* = 7,018,172)^a^	Study population (%; *n* = 2600)
Sex		
Men	49.6	49.8
Women	50.4	50.2
Age (years)		
19–29	20.7	22.7
30–39	24.0	26.0
40–49	24.5	26.6
≥ 50	30.8	24.7
Region		
Urban areas	5.1	5.0
Northeast	31.2	31.4
Northwest	11.8	11.7
Southeast	30.5	30.4
Southwest	21.4	21.6

^a^The *Statistics* of the Registered Population in August, 2015 [18]

The relationship of socio-demographic factors with the frequency of SHS incursion for smoking and non-smoking residents is shown in Table 2. A total of 74.8% of the residents were non-smokers. Women were more likely than men to be non-smokers (62.1%, *p* < 0.001). Non-smokers were older (*p* < 0.001) and had lower household incomes (*p* = 0.035) compared with smokers. Non-smokers were more likely than smokers to live in an apartment (51.2%, *p* < 0.001) and to spend more time at home (*p* < 0.001), and were less likely to live with children (38.6%, *p* = 0.018). Non-smokers were likely to have been residents for a longer period (*p* = 0.019). Non-smokers were more likely than smokers to support the implementation of smoke-free regulations in MUH (89.9%, *p* < 0.001), and to live in homes with a personal smoke-free rule (72.1%, *p* < 0.001). Non-smokers were more likely than smokers to have reported an SHS incursion within the previous 12 months (*p* < 0.001). However, level of educational attainment, number of residents, type of ownership, and presence of other smokers inside a home did not differ between smoking and non-smoking residents.

**Table 2:** Characteristics between smoking and non-smoking resident in MUH

	Total (%; *n* = 2600)	Smoker (%; *n* = 654)	Non-smoker (%; *n* = 1946)	*p*-value
Overall	100.0	25.2	74.8	
Sex				
Men	49.8	85.2	37.9	<0.001
Women	50.2	14.8	62.1	
Age (years)				
19–29	22.7	19.6	23.7	<0.001
30–39	26.0	28.6	25.2	
40–49	26.6	31.2	25.1	
≥ 50	24.7	20.6	26.0	
Household income (USD/month)				
< 2000	7.2	5.7	7.8	0.035
2000–3999	29.2	28.1	29.5	
4000–5999	37.0	35.9	37.4	
6000–7999	15.6	16.5	15.3	
≥ 8000	11.0	13.8	10.0	
Education				
Less than university level	34.9	33.2	35.5	0.418
University level	56.2	58.4	55.4	
More than university level	8.9	8.4	9.0	
Housing type				
Apartment	50.1	46.8	51.2	<0.001
Attached home	49.9	53.2	48.8	
Time spent at home (hours/day)				
< 5	27.5	35.6	24.8	<0.001
5–9	51.8	51.7	51.8	
≥ 10	20.7	12.7	23.4	
Number of residents (people)				
< 4	49.2	52.4	48.2	0.057
≥ 4	50.8	47.6	51.8	
Children living in home (aged ≤18 years)				
No	60.0	56.1	61.4	0.018
Yes	40.0	43.9	38.6	
Type of ownership				
Owned	56.7	54.1	57.6	0.179
Leased based on deposit	29.7	30.4	29.4	
Monthly rent	13.6	15.4	12.9	
Duration of residence (years)				
< 2	26.9	25.4	27.4	0.019
2–3	19.8	23.5	18.5	
≥ 4	53.3	51.1	54.1	
Presence of other smokers inside the home^a^				
No	62.9	60.2	63.8	0.819
Yes	37.1	39.8	36.2	
Support for the implementation of smoke-free regulations in MUH				
No	16.8	37.0	10.1	<0.001
Yes	83.2	63.0	89.9	
Living in a home with a personal smoke-free rule				
No	37.5	66.2	27.9	<0.001
Yes	62.5	33.8	72.1	
Frequency of SHS incursion				
Never	28.6	37.2	25.7	<0.001
≤ 1 times/month	19.4	16.5	20.3	
2–4 times/month	25.3	25.2	25.3	
> 4 times/month	26.8	21.1	28.7	

^a^Smokers among family members or regular visitors to the home

The proportions of general smoking locations in smokers’ homes were estimated using data from residents who were either smokers or resided with smokers (*n* = 1359). Among the residents who smoked at their homes (*n* = 560), the most common smoking location was the balcony (51.4%, *n* = 288), followed by the bathroom (20.2%, *n* = 113), main room (14.8%, *n* = 83), and outside the front door (13.6%, *n* = 76).

Although there was no difference in SHS incursion between the non-smoking residents who lived in homes with and without a personal smoke-free rule (*p* = 0.568), only non-smoking residents who lived in homes with a personal smoke-free rule were used for further analysis (*n* = 1784). In total, 74.7% of these non-smoking residents (*n* = 1333) reported that they had experienced SHS incursion into their home within the previous 12 months. In total, 9.9% of the residents (*n* = 176) reported that they had experienced SHS incursion every day, and 44.2% (*n* = 788) reported that they had experienced SHS incursion once a week or more. The residents who had experienced SHS incursion reported the entry point of SHS into their homes (*n* = 1333); the main source of SHS incursion was the balcony (45.7%, *n* = 609), followed by windows (28.4%, *n* = 378), bathroom (12.9%, *n* = 172), front door (11.7%, *n* = 156), and other locations (1.4%, *n* = 18).

Table 3 shows characteristics of the non-smoking residents living in home with a personal smoke-free rule by frequency of SHS incursion. Residents who were women (*p* = 0.020), spent more time at home (*p* < 0.001), lived with children (*p* < 0.001), and supported the implementation of smoke-free homes in MUH (*p* = 0.020) exhibited a positive trend across the categories of SHS incursion. Residents who lived in large homes exhibited a negative trend across the categories (*p* = 0.038). Method of natural ventilation at residents’ homes was related to frequency of SHS incursion (*p* = 0.042). Residents who lived in homes with more frequent natural ventilation exhibited a positive trend across the categories (*p* < 0.001). However, age, household income, level of educational attainment, housing type, number of residents, type of ownership, duration of residential period, presence of other smokers inside the home, date of construction, type of corridor, presence of a balcony, and air-conditioning were not significantly associated with frequency of SHS incursion.

**Table 3:** Characteristics of the non-smoking residents living in home with smoke-free rules by SHS incursion

	Total (%; *n* = 1784)	Frequency of SHS incursion				
		Never (%, *n* = 451)	≤1 times/month (%, *n* = 366)	2–4 times/month (%, *n* = 452)	>4 times/month (%, *n* = 515)	*p*-value^a^
Socio-demographic factor						
Sex						
Men	38.5	39.9	42.6	39.8	33.2	0.020
Women	61.5	60.1	57.4	60.2	66.8	
Age (years)						
19–29	22.6	24.4	20.2	23.7	21.9	0.079
30–39	26.0	23.1	23.5	29.2	27.6	
40–49	24.8	20.4	28.1	23.9	27.0	
≥ 50	26.6	32.2	28.1	23.2	23.5	
Household income (USD/month)						
< 2000	7.8	10.4	6.0	5.1	9.3	0.171
2000–3999	29.6	30.6	29.2	29.9	28.7	
4000–5999	36.7	37.5	36.3	37.8	35.3	
6000–7999	15.2	11.1	18.6	16.8	15.1	
≥ 8000	10.6	10.4	9.8	10.4	11.5	
Education						
Less than university level	34.8	37.3	30.1	33.6	36.9	0.698
University level	55.5	54.5	57.9	54.6	55.5	
More than university level	9.7	8.2	12.0	11.7	7.6	
Housing type						
Apartment	51.6	50.1	57.9	53.8	46.6	0.147
Attached house	48.4	49.9	42.1	46.2	53.4	
Time spent at home (hours/day)						
< 5	25.3	29.5	27.3	25.7	20.0	<0.001
5–9	51.7	52.3	50.0	52.2	52.0	
≥ 10	22.9	18.2	22.7	22.1	28.0	
Number of residents (people)						
< 4	49.0	51.9	49.7	46.2	48.5	0.207
≥ 4	51.0	48.1	50.3	53.8	51.5	
Children living in home (aged ≤18 years)						
No	61.0	70.1	59.0	58.4	56.9	<0.001
Yes	39.0	29.9	41.0	41.6	43.1	
Type of ownership						
Owned	57.4	56.5	59.0	59.3	55.3	0.830
Leased based on deposit	29.9	27.9	30.1	29.9	31.5	
Monthly rent	12.7	15.5	10.9	10.8	13.2	
Duration of residence (years)						
< 2	27.8	30.4	24.6	24.1	31.1	0.495
2–3	18.7	17.7	16.9	20.6	19.2	
≥ 4	53.5	51.9	58.5	55.3	49.7	
Presence of other smokers inside home^b^						
No	69.6	72.9	71.0	64.8	69.7	0.116
Yes	30.4	27.1	29.0	35.2	30.3	
Support for the implementation of smoke-free regulations in MUH						
No	9.0	12.4	6.8	9.3	7.2	0.020
Yes	91.0	87.6	93.2	90.7	92.8	
Built environmental factor						
Date of construction (year)						
Before 1995	27.3	27.3	26.8	24.8	29.9	0.322
1995–1999	23.5	23.7	22.4	24.8	22.9	
2000–2004	22.0	21.5	22.4	23.2	21.2	
2005–2009	14.7	12.6	18.0	13.5	15.1	
2010 or later	12.5	14.9	10.4	13.7	10.9	
Type of corridor						
Stairwell	77.1	78.7	78.4	75.0	76.7	0.142
Indoor corridor	14.0	14.2	13.4	15.0	13.4	
Outdoor corridor	8.9	7.1	8.2	10.0	9.9	
Home size (m^2^)						
< 66	25.2	26.8	22.7	22.1	28.3	0.038
66–98	35.8	32.6	32.5	38.1	39.0	
≥ 99	39.0	40.6	44.8	39.8	32.6	
Presence of balcony						
No	22.1	23.3	25.4	19.9	20.6	0.128
Yes	77.9	76.7	74.6	80.1	79.4	
Presence of air–conditioning						
No	18.3	19.5	16.9	14.6	21.6	0.558
Yes	81.7	80.5	83.1	85.4	78.4	
Method of natural ventilation						
Opening windows	52.7	55.4	58.5	50.0	48.7	0.042
Opening front doors	5.3	4.2	4.4	6.4	6.0	
Opening windows and front doors	18.3	16.2	14.8	22.1	19.2	
Windows always slightly open	23.7	24.2	22.4	21.5	26.0	
Frequency of natural ventilation (times/week)						
< 5	31.9	34.4	38.5	32.7	24.3	<0.001
≥ 5	68.1	65.6	61.5	67.3	75.7	

^a^The Cochran-Mantel-Haenszel test

^b^Smokers among family members or regular visitors to the home

The univariate and multivariate ordinal logistic regression model of SHS incursion are shown in Table 4. In the univariate analysis, the proportional odds assumption was violated for home size (*p* = 0.049) and frequency of natural ventilation (*p* = 0.019); thus, different effects in these variables were estimated for the different levels of frequency of SHS incursion. In the multivariate analysis, all variables except sex seemed consistent effects with univariate analysis on SHS incursion. Residents who spent 5–9 h/day and those who spent ≥10 h/day at home were more likely to report SHS incursion than were those who spent <5 h/day at home. Residents who lived with children and those who supported the implementation of smoke-free regulations in MUH were more likely to report SHS incursion than were those who did not. Residents who lived in home sized ≥99 m^2^ were less likely to report SHS incursion in the 2 highest SHS incursion categories and in the highest SHS incursion categories than were those who lived in home sized <66 m^2^. Residents who lived in homes with natural ventilation provided by open front doors or both open windows and front doors were more likely to report SHS incursion than were those with only open windows. Residents who lived in homes with a natural ventilation frequency of ≥5 times/week were more likely to report SHS incursion in the 3 and 2 highest SHS incursion categories and in the highest SHS incursion categories than were those who lived in homes with ventilation frequency of <5 times/week.

**Table 4:** Factors associated with SHS incursion among non-smoking residents living in home with smoke-free rules^a^

	Univariate		Multivariate	
	OR (95% CI)^b^	*p*-value	OR (95% CI)^c^	*p*-value
Socio-demographic factor				
Sex				
Men	1.00		1.00	
Women	1.23 (1.04–1.46)	**0.018**	1.11 (0.93–1.33)	0.242
Time spent at home (hours/day)				
< 5	1.00		1.00	
5–9	1.29 (1.05–1.58)	**0.014**	1.28 (1.05–1.57)	**0.017**
≥ 10	1.68 (1.32–2.14)	**<0.001**	1.60 (1.25–2.06)	**<0.001**
Children living in home (aged ≤18 years)				
No	1.00		1.00	
Yes	1.41 (1.19–1.67)	**<0.001**	1.40 (1.17–1.66)	**<0.001**
Support for the implementation of smoke-free regulations in MUH				
No	1.00		1.00	
Yes	1.43 (1.07–1.91)	**0.017**	1.47 (1.09–1.99)	**0.011**
Built environmental factor				
Home size (m^2^)				
< 66	1.00		1.00	
66–98				
OR 1^d^	1.23 (0.93–1.63)	0.730	1.15 (0.87–1.53)	0.327
OR 2^e^	1.16 (0.91–1.48)	0.224	1.05 (0.82–1.35)	0.690
OR 3^f^	0.96 (0.74–1.24)	0.143	0.87 (0.67–1.13)	0.290
≥ 99				
OR 1^d^	1.03 (0.79–1.35)	0.835	0.95 (0.72–1.25)	0.695
OR 2^e^	0.83 (0.66–1.06)	0.129	0.73 (0.57–0.94)	**0.013**
OR 3^f^	0.66 (0.51–0.86)	**0.002**	0.59 (0.45–0.77)	**<0.001**
Method of natural ventilation				
Opening windows	1.00		1.00	
Opening front doors	1.46 (1.00–2.14)	0.051	1.59 (1.08–2.33)	**0.018**
Opening windows and front doors	1.32 (1.05–1.65)	**0.017**	1.27 (1.01–1.60)	**0.038**
Windows always slightly open	1.17 (0.96–1.44)	0.128	1.17 (0.95–1.45)	0.137
Frequency of natural ventilation (times/week)				
< 5	1.00		1.00	
≥ 5				
OR 1^d^	1.16 (0.93–1.46)	0.193	1.11 (0.88–1.40)	**<0.001**
OR 2^e^	1.44 (1.18–1.76)	**<0.001**	1.41 (1.15–1.74)	**0.001**
OR 3^f^	1.68 (1.33–2.12)	**<0.001**	1.64 (1.29–2.08)	**<0.001**

ORs with *p* < 0.05 are in bold

^a^Cumulative logistic models were used when the proportion odds assumption were retained and partial proportional odds models were used when the assumption was violated. Proportional odds assumption is violated for home size (*p* = 0.049) and frequency of natural ventilation (*p* = 0.019) but others were met the assumption (*p* > 0.05)

^b^Unadjusted OR

^c^Adjusted OR: adjusted for all variables listed in the table

^d^OR 1: >4, 2–4, or ≤1 times/month vs. never

^e^OR 2: >4 or 2–4 times/month vs. ≤1 times/month or never

^f^OR 3: >4 times/month vs. 2–4 or ≤1 times/month or never

We further conducted an ordinal logistic regression analysis among smoking residents living in home with a smoke-free rule (*n* = 433). The final multivariate ordinal logistic regression model was fit for the proportional odds assumption (*p* = 0.179). In the multivariate analysis, residents who lived with children (OR = 1.53, 95% CI = 1.07–2.17) and those who supported the implementation of smoke-free regulations in MUH (OR = 2.07, 95% CI = 1.34–3.18) were more likely to report SHS incursion than were those who did not. Residents who lived in homes with indoor corridor (OR = 1.65, 95% CI = 1.04–2.63) and those who lived in homes with natural ventilation provided by both windows and front doors (OR = 2.65, 95% CI = 1.70–4.12) or those with always slightly open windows (OR = 2.13, 95% CI = 1.34–3.39) were more likely to report SHS incursion than a reference value. Other variables were not significantly associated with SHS incursion.

## Discussion

The smoking rate of MUH residents in the study population was 25.2%, which was higher than that in the Seoul general population in 2014. Based on statistical data from the Community Health Survey (CHS), a comprehensive health status survey program in Korea, the smoking rate in the Seoul population (≥19 years) in 2014 was 20.6% [22]. The results of the CHS indicate that the smoking rate increases with age from 19 to 49 years (20.3–25.8%), but then decreases sharply from 50 to 70 years or older (9.0–13.9%). One possible reason for the higher smoking rate in this study could be the low proportion of respondents older than 60 years, which might have led to an overestimation of the smoking rate.

The self-reported frequency of SHS incursion differed between smoking and non-smoking residents. In the present study, smokers were less likely to report SHS incursion. This might be explained by a difference in the perception of SHS exposure between smokers and non-smokers. Smokers could be habituated and less likely to be irritated by to the smell of SHS [16]. Similar findings have been reported that residents who were smokers were less likely to report SHS incursion in MUH than were non-smokers [13, 15, 16].

Among the non-smokers who lived in homes with a personal smoke-free rule, 74.7% had experienced SHS incursion within the previous 12 months. One in 10 residents reported that they experienced daily SHS incursion. The prevalence of SHS incursion in this study was higher than that reported in previous studies. In a 2010 study in the US, 44% of residents in MUH with a personal smoke-free home rule had experienced SHS incursion in their units within the previous 12 months [23]. In that study, the smoking rate of the residents was 21.1%. In a 2009 study in New York State, 46.2% of residents with a personal smoke-free home policy had experienced SHS incursion in their unit within the previous 12 months [15]. The smoking rate of the study population was 19.0%. A possible reason for the high prevalence of SHS incursion in the present study might be because smoking rate in this study was higher than that in previous studies conducted in the USA.

The majority of non-smoking residents who had experienced SHS incursion within the past 12 month reported that SHS entered their homes through the balcony or windows. The ingress route taken by SHS incursion was slightly higher in bathrooms than through the front door. SHS could migrate through the balcony [15], hallway (similar to a corridor) [11], and bathroom ceiling exhaust fans [24]. In this study, it was suggested that SHS incursion into bathrooms might have been associated with migration of SHS through bathroom ceiling exhaust fans in other units. A front door was associated with migration of SHS from the corridor outside a home.

In this study, the source of SHS incursion was consistent with the smoking locations used by smokers in their homes in MUH. The most common smoking location was the balcony, followed by the bathroom, main room, and outside the front door. This suggested that smoking in these locations might be associated with SHS incursion into other units. Therefore, limitations on smoking in these locations should be placed to reduce the SHS incursion into other units in MUH. Because it might be difficult to implement smoke-free regulations in MUH, offering educational information on how to implement smoke-free policy to building managers or owners could be the first step for smoke-free MUH [25].

In the multivariate analysis, residents who spent more time at home were more likely to report SHS incursion. As the time spent at home increased, the ORs of SHS incursion also tended to increase. As residents spend more time in their home, they are more likely to be exposed to SHS incursion. Thus, MUH residents who spend long periods at home might be at risk of high SHS exposure from such incursion.

Residents who lived with children and who supported the implementation of smoke-free regulations in MUH were more likely to report SHS incursion. MUH residents who lived with children might be more sensitive to SHS incursion because their children are being exposed to SHS [13]. MUH residents who experienced a high level of SHS incursion might express more support for smoke-free regulations in MUH so as to reduce their SHS exposure at home.

Among the built environmental factors investigated here, home size was significantly associated with SHS incursion. Overall, residents who lived in homes ≥99 m^2^ in size were less likely to report SHS incursion than were those in homes of <66 m^2^. This might be because home size was associated with housing type. In Korea, the average home size per person was larger in an apartment than in an attached home in 2010 [26]. In the present study, residents who lived in an apartment were slightly less likely to report SHS incursion than were those in an attached home. Therefore, residents who lived in larger homes were more likely to live in an apartment and might therefore be less likely to experience SHS incursion.

Factors related to natural ventilation were associated with SHS incursion. Residents who lived in homes with natural venation provided by opening the front door or by opening both the front door and windows were more likely to report SHS incursion than were those with natural venation provided only by opening the windows. The ORs for providing natural ventilation with an open front door were higher than those where natural ventilation was provided by opening both windows and front doors. Furthermore, residents who frequently used natural ventilation were more likely to report SHS incursion. The results of the study indicate that residents who lived in homes where natural ventilation was provided by opening the front door and those who lived in homes with frequent natural ventilation were more likely to be exposed to SHS incursion.

In this study, SHS incursion, a dependent variable, was assigned as an ordinal variable in a logistic regression analysis. Previous studies have used dichotomized dependent variables for SHS incursion to examine associated factors [15, 23]. When we used SHS incursion as a dichotomized dependent variable (i.e., no = 0 vs. yes = 1), the factors associated with SHS incursion among non-smoking residents living in home with a smoke-free rule in the multivariate logistic regression analysis were household income, children living in the home, time spent at home, and support for the implementation of smoke-free regulations in MUH. Other variables were not significantly associated with SHS incursion. This indicated that using SHS incursion as an ordinal variable might be a more useful approach to examine predictors for SHS incursion in MUH.

To our knowledge, this is the first study to determine prevalence and predictors of SHS incursion among MUH residents in Korea. The present study included socio-demographic factors as well as built environmental factors to determine predictors on SHS incursion. The findings of the present study could be useful for targeted effort to promote smoke-free regulation in MUH and understanding SHS exposure of residents in homes due to SHS incursion.

This study has a few limitations. We used self-reported SHS incursion experienced by residents within the previous 12 months. The self-report measure might be subject to variations and recall-bias due to a respondent’s sensitivity. Because SHS incursion was less likely to be reported by residents who were smokers, we used data from non-smoking residents to identify the factors associated with SHS incursion, which enabled better estimations. Another limitation was that SHS incursion was based on the detection of SHS by smell by MUH residents. Because we measured SHS incursion using a self-reported questionnaire, we could not confirm or quantify each resident’s exposure to SHS due to SHS incursion. Furthermore, self-report of SHS might partially be due to third-hand smoke particularly for the home with smokers in the past. Further study is needed using more specific SHS markers to provide a better understanding of SHS incursion in MUH.

## Conclusions

A sample of 2600 MUH residents in Seoul, Korea, was investigated. The majority of non-smoking respondents who lived in homes with a personal smoke-free rule experienced SHS incursion in their units within the previous 12 months. The high prevalence of SHS incursion suggests that most residents might be at risk from exposure due to SHS incursion. SHS incursion was associated with time spent at home, living with children, support for the implementation of smoke-free regulations in MUH, home size, and the method and frequency of natural ventilation used. Built environmental factors identified in the study could be useful to understand exposure due to SHS incursion at homes in MUH. Smoke-free policies in MUH are needed to protect MUH residents from SHS exposure in their homes.

## Additional file

Additional file 1: Table S1. Fourteen items of socio-demographic information. Table S2. Two items of smoking status. Table S3. Two items of secondhand smoke incursion at home. Table S4. Seven items of built environmental information. (DOCX 26 kb)

## Abbreviations

CHS: Community Health Survey
CI: Confidence interval
MI: Ministry of the Interior
MUH: Multi-unit housing
OR: Odds ratio
SHS: Secondhand smoke
