# Supplementary material for: Factors associated with secondhand smoke incursion into the homes of non-smoking residents in a multi-unit housing complex: a cross-sectional study in Seoul, Korea
Source: BMC Public Health. 2017 Sep 25;17:739. doi: 10.1186/s12889-017-4774-x (PMC5613333; doi:10.1186/s12889-017-4774-x)
Supplement: Additional file 1: Table S1. — Fourteen items of socio-demographic information. Table S2. Two items of smoking status. Table S3. Two items of secondhand smoke incursion at home. Table S4. Seven items of built environmental information. (DOCX 26 kb) [file 12889_2017_4774_MOESM1_ESM.docx]

**Additional files 1.**

**Table S1. Fourteen items of socio-demographic information**

| D1. What type of housing do you live in? | 🞏 Apartment  🞏 Attached house  🞏 Detached house  🞏 Others |
| --- | --- |
| D2. What is your age now? | ________________ |
| D3. In which region do you live? | ________________ |
| D4. What is your gender? | 🞏 Male  🞏 Female |
| D5. What is the average monthly income of households (combining myself and family)? | 🞏 less than $2,000  🞏 $2,000-$3,999  🞏 $4,000-$5,999  🞏 $6,000-$7,999  🞏 $8,000 or more |
| D6. What is the highest level of education you have completed? | 🞏 School  🞏 College  🞏 University  🞏 Postgraduate |
| D7. How many hours do you usually stay at homes except for sleeping time? | 🞏 less than 2 hours  🞏 2-4 hours  🞏 5-9 hours  🞏 10-14 hours  🞏 15 hours or more |
| D8. How many people are currently living in your household, including yourself? | 🞏 1 person  🞏 2 people  🞏 3 people  🞏 4 people  🞏 5 people or more |
| D9. How many children (18 years old or less) are currently living in your household | 🞏 None  🞏 1 person  🞏 2 people  🞏 3 people or more |
| D10. What type of ownership in your home is? | 🞏 Owned  🞏 Leased based on deposit  🞏 Monthly rent |
| D11. How long have you been in your home? | 🞏 Less than 1 year  🞏 1 year or more but less than 2 years  🞏 2 year or more but less than 4 years  🞏 4 years or more |
| D12. Do you agree that smoke-free regulations should be implemented at homes in multi-unit housing? | 🞏 No  🞏 Yes |
| D13. Do you live in the same household with someone who smokes tobacco? | 🞏 There were smokers among the family  🞏 There were smokers among regular guests or friends  🞏 None |
| D14. Which of following statement best described the rules about smoking in your home? | 🞏 Smoking is not allowed anywhere inside home  🞏 Smoking is allowed in some places or at some times  🞏 Smoking is allowed anywhere in the room |

**Table S2. Two items of smoking status**

| S1. Are you currently smoking? | 🞏 No, never  🞏 No, in the past but not currently  🞏 Yes, sometimes  🞏 Yes, every day |
| --- | --- |
| S2. If there are smokers in your home, where do they mainly smoke? | 🞏 Balcony  🞏 Main room  🞏 Bathroom  🞏 Just outside the front door  🞏 Outside the building  🞏 Others |

**Table S3. Two items of secondhand smoke incursion at home**

| I1. How often could you smell tobacco smoke that entered your living space from somewhere else in or around your building during a 12-month period | 🞏 Never  🞏 Once a month or less  🞏 Twice a month  🞏 Four times a month  🞏 Two to four times a week  🞏 Every day |
| --- | --- |
| I2. Where had the secondhand smoke entered into your home? | 🞏 Balcony  🞏 Window  🞏 Bathroom  🞏 Front door  🞏 Other location |

**Table S4. Seven items of built environmental information**

| B1. When the multi-unit housing where you reside was built? | 🞏 Before 1995 year  🞏 1995-1999 year  🞏 2000-2004 year  🞏 2005-2009 year  🞏 2010 or later |
| --- | --- |
| B2. What type of corridor in the multi-unit housing where you reside is? | 🞏 Stairwell  🞏 Indoor corridor  🞏 Outdoor corridor |
| B3. How large is your home? | 🞏 less than 66 m^2^  🞏 66-98 m^2^  🞏 99-131 m^2^  🞏 132 m^2^ or more |
| B4. Is there a balcony in your home? | 🞏 No  🞏 Yes |
| B5. Is there an air conditioning in your home? | 🞏 No  🞏 Yes |
| B6. How do you usually ventilate by opening window or front door in your home? | 🞏 Opening windows facing outdoors  🞏 Opening the front door  🞏 Opening both windows facing outdoor and front doors  🞏 Always slightly opening windows facing outdoors |
| B7. How often do you usually ventilate by opening window or front door in your home? | 🞏 less than 1 times/week  🞏 1-2 times/week  🞏 3-4 times/week  🞏 5 times/week or more |
